# HIV-2 glycoproteins upregulate microRNAs 25 and 93 to counter the MARCH1 antiviral effect in macrophages

**DOI:** 10.1128/jvi.01663-25

**Published:** 2025-11-24

**Authors:** Robert Lodge, Dorota Kmiec, Frank Kirchhoff, Éric A. Cohen

**Affiliations:** 1 Laboratory of Human Retrovirology, Institut de recherches cliniques de Montréal (IRCM) 5598, Montreal, Quebec, Canada; 2 Institute of Molecular Virology, Ulm University Medical Center 27197, Ulm, Germany; 3 Department of Microbiology, Infectious Diseases and Immunology, Université de Montréal 5622, Montreal, Quebec, Canada; The Ohio State University, Columbus, Ohio, USA

**Keywords:** microRNA, MARCH1, host restriction factor, HIV-2, SIV, Env glycoprotein, beta-catenin, beta-TrCP, macrophage, THP-1

## Abstract

The membrane-associated E3 ubiquitin ligase MARCH1 restricts HIV-1 infectivity by decreasing the amount of cell surface Env glycoproteins and their incorporation into progeny virions. To circumvent this restriction, the HIV-1 accessory protein Vpu increases the levels of microRNAs 25 and 93 that target *MARCH1* RNA. Mechanistically, Vpu interaction with the β-TrCP substrate receptor of the SCF^β-TrCP^ E3 ligase, a host partner critical for Vpu functions, leads to a stabilization of SCF^β-TrCP^ cellular targets, such as β-catenin, which drives transcription of the *MARCH1*-targeting microRNAs. Here, we show that HIV-2 and SIVmac239, which do not encode for Vpu, also upregulate microRNAs 25 and 93 in macrophages, as well as in the macrophage-like THP-1 cell line model. Inhibiting the *MARCH1* mRNA-targeting microRNAs impaired HIV-2 infectivity and reduced viral spread in macrophages. While none of the HIV-2 accessory proteins upregulated microRNAs 25 and 93, the Env glycoproteins of HIV-2 and SIVmac239 were sufficient to induce their expression. Depletion of β-TrCP-1 and 2 or pharmacological inhibition of β-catenin in THP-1 cells revealed that Env-mediated upregulation of microRNA 25 and 93 is β-TrCP dependent and involves β-catenin, similar to Vpu. Our findings highlight a new function of the HIV-2 and SIVmac239 glycoproteins as inducers of microRNAs 25 and 93 that counteract MARCH1 and potentiate viral replication in macrophages.

**IMPORTANCE:**

Macrophage infection by primate lentiviruses (HIV-1, HIV-2, and SIV) is restricted by, among other host factors, the MARCH1 membrane-associated protein. HIV-1 circumvents the MARCH1 restriction by using its accessory protein Vpu, which induces the anti-MARCH1 microRNAs 25 and 93. HIV-2 has infected up to 2 million people so far, and SIV infection is indispensable for animal models of primate lentivirus pathogenesis. These two lentiviruses do not have a *vpu* gene, but also target MARCH1 by inducing the same microRNAs. We have now determined that the HIV-2 and SIV antagonists of MARCH1 are their envelope glycoproteins. The HIV-2 and SIVmac239 Env glycoproteins upregulate microRNAs 25 and 93 by disrupting the β-catenin pathway, similar to HIV-1 Vpu. The fact that different lentiviruses have developed a similar strategy using microRNAs to counter MARCH1 antiviral activity emphasizes its relevance in the antiviral host response, as well as a target in antiviral therapy.

## INTRODUCTION

In order to counter viral infection, replication, and spread, mammalian cells have developed several antiviral defenses that often target specific steps of the virus replicative cycle. Some of these antiviral factors target the viral envelope glycoproteins, which mediate virus entry into the host cell (1). Among them are members of the membrane-associated RING (really interesting new gene)-CH (MARCH) protein family of E3 ubiquitin ligases (2). Besides having important roles in the cell, such as the plasma membrane turnover and trafficking of MHC class II molecules (3), MARCH proteins inhibit the incorporation of viral envelope glycoproteins into nascent virions (4, 5). Indeed, MARCH proteins restrict the viral glycoproteins of several enveloped viruses, including vesicular stomatitis virus (VSV), influenza virus, lymphocytic choriomeningitis virus, Chikungunya virus, Ebola virus, and severe acute respiratory syndrome coronavirus 2 (6–9).

MARCH1, 2, and 8 proteins have also been shown to restrict HIV-1 Env glycoprotein incorporation, although by different mechanisms depending on cell types and conditions (7–11). For example, although restriction through MARCH8 requires a direct interaction and ubiquitination of the cytoplasmic tail (CT) of VSV-G glycoprotein (7, 10), this is not the case for HIV-1 Env (7, 10). Recently, MARCH2 was reported to restrict HIV-1 glycoproteins specifically in CD4^+^ T cells, but not in monocyte-derived macrophages (MDMs) (12). Unlike the ubiquitously expressed MARCH2 and 8 (13), MARCH1 is mostly found in myeloid cells, such as MDMs and dendritic cells (5, 14), and is induced by type I interferon (IFN-I) in MDMs (14). In HIV-1-infected macrophages, MARCH1 restricts the incorporation of HIV-1 glycoproteins into nascent virions, a condition that results in impairment of HIV-1 infectivity and spread (14). In order to do this, MARCH1 redirects the HIV-1 glycoproteins to a perinuclear region, reducing their presence at the cell surface and availability to budding viruses (14).

In order to counter cellular antiviral restriction factors, HIV-1 encodes the accessory proteins Vif, Nef, Vpr, and Vpu (15, 16). Vpu antagonizes the IFN-I inducible restriction factor BST2 (also called tetherin) to promote efficient release of virions (17, 18), and targets the CD4 receptor to favor newly synthesized Env glycoprotein transport to the cell surface and infectivity of progeny virions (19–21). The mechanism by which Vpu targets these host factors is by exploiting the SCF (Skp1/Cullin1/F-box protein)-β-TrCP (β-transducin repeat-containing protein) E3 ubiquitin ligase, recruiting this complex, via β-TrCP binding, to ubiquitinate and direct Vpu target proteins for degradation (22, 23). The recruitment of β-TrCP by Vpu also brings an increase in β-TrCP’s other host targets, notably β-catenin and IκB, thus affecting the expression of genes regulated by these pathways (24). This increase of β-catenin upregulates several host microRNAs (14, 25, 26), among which are microRNAs 25 and 93 that downregulate target *MARCH1* mRNA (14). HIV-2, of which the infection affects 2 million people (27), and SIVmac239, which is commonly used in non-human primate models of HIV/AIDS, are lentiviruses that also express accessory proteins to counteract host restriction factors (28). In addition to Vif, Nef, and Vpr, HIV-2 and SIV encode the accessory Vpx protein, which allows them to counteract the host restriction factor sterile alpha motif and HD domain-containing protein 1 (SAMHD1) by inducing its proteasomal degradation (29, 30). Indeed, SAMHD1 reduces intracellular dNTP concentrations, which are important for retrovirus reverse transcription and replication, particularly in non-dividing cells (31, 32). Finally, both HIV-2 and most SIVs do not encode for a Vpu protein (28). Recently, we showed that, despite the lack of Vpu, HIV-2 and SIVmac239 upregulate microRNAs 25 and 93 in infected macrophages and reduce the levels of *MARCH1* mRNA (14) by a yet undefined mechanism.

Here, we report a new function of the HIV-2 and SIV glycoproteins as inducers of microRNAs 25 and 93 and thus regulators of *MARCH1* mRNA in infected macrophages. Inhibiting the *MARCH1* mRNA-targeting microRNAs impaired HIV-2 spread by preventing MARCH1 downregulation in infected macrophages. Upregulation of microRNAs 25 and 93 required *de novo* Env glycoprotein expression and was dependent on β-catenin and regulated by the β-TrCP ubiquitin ligase.

## RESULTS

### MicroRNAs 25 and 93 are upregulated in HIV-2 and SIVmac-infected macrophages and enhance HIV-2 infection

**Fig 1:**
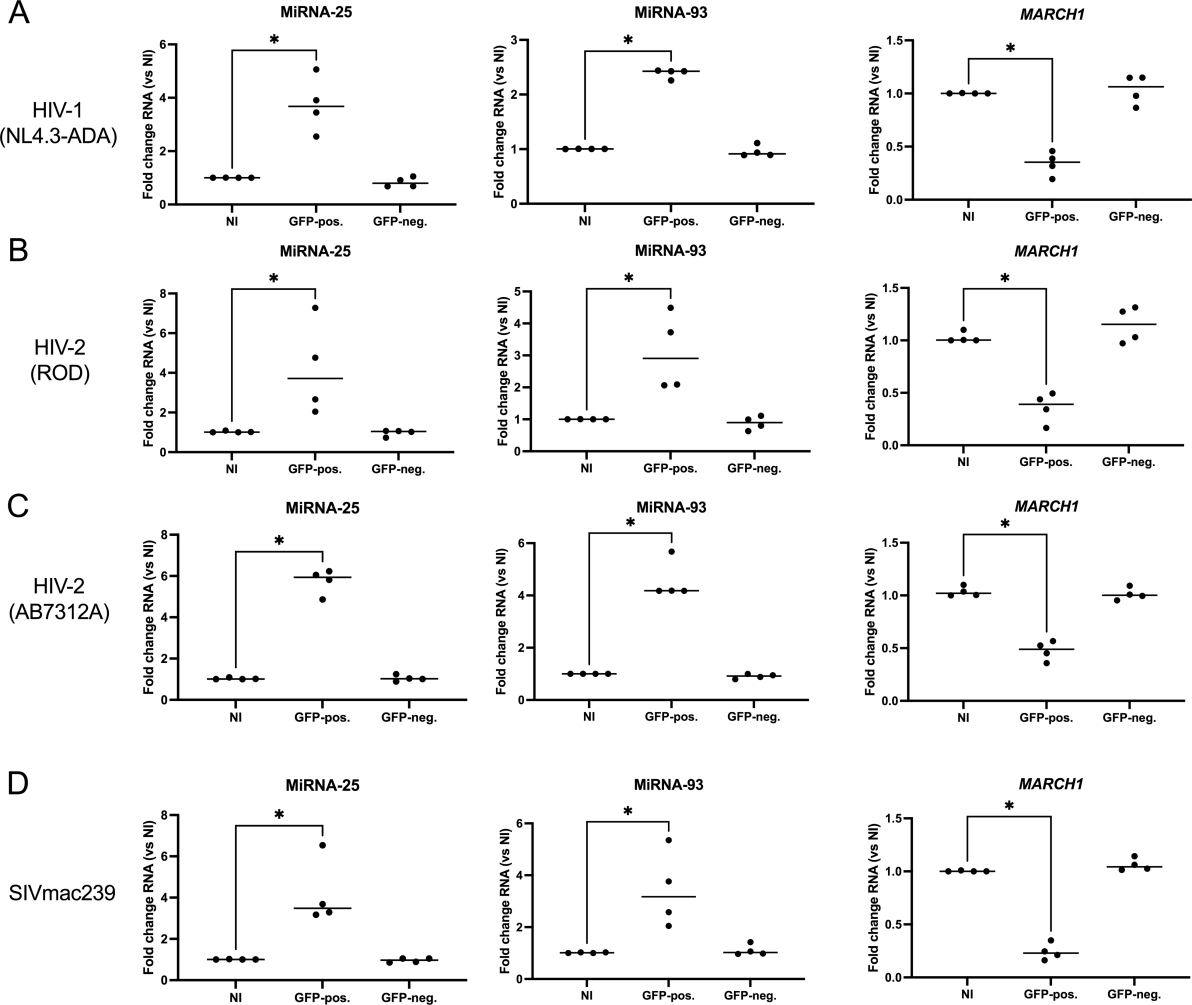
MicroRNAs 25 and 93 are upregulated, whereas *MARCH1* mRNA is reduced, in HIV-1, HIV-2, and SIV-infected human MDMs. MDMs from four different blood donors were infected with the indicated VSV-G pseudotyped GFP-expressing viruses for 36 h and then sorted. Total RNAs of the GFP+/GFP− cells were extracted, reverse-transcribed, and microRNAs 25 and 93, as well as *MARCH1* mRNA, quantified by qRT-PCR. Fold changes are compared to uninfected cells (NI). (**A**) HIV-1 NL4.3-ADA. (**B**) HIV-2 ROD. (**C**) HIV-2 AB7312A. (**D**) SIVmac239. *P < 0.05, using the Mann-Whitney U test.

In order to investigate the mechanisms involved in Vpu-independent microRNA 25 and 93 upregulation by HIV-2/SIVmac in infected macrophages (14), we first confirmed the increased expression of microRNAs 25 and 93, as well as the reduction of *MARCH1* mRNA, in human MDMs infected 36 h with either the HIV-2 AB7312A strain (33) or the previously reported HIV-1 (NL4.3-ADA), HIV-2 (ROD), or SIVmac239 viruses (14). These viruses were modified to additionally express GFP, enabling cell sorting into infected and bystander cell populations (Fig. S1). Total RNAs were extracted from both infected and bystander MDMs, and the levels of microRNAs 25 and 93, as well as *MARCH1* mRNA, were determined by quantitative reverse transcription-PCR (qRT-PCR). As previously reported (14), we observed an increase in the levels of microRNAs 25 and 93 (as compared to uninfected controls), as well as a reduction in the amount of *MARCH1* mRNA, in the GFP+ population of either HIV-1, HIV-2, or SIV infected cells (Fig. 1). The increase was observed in both cell-line passaged ROD strain, as well as patient-derived primary isolate AB7312A, suggesting that this phenotype is shared by epidemic HIV-2 strains.

**Fig 2:**
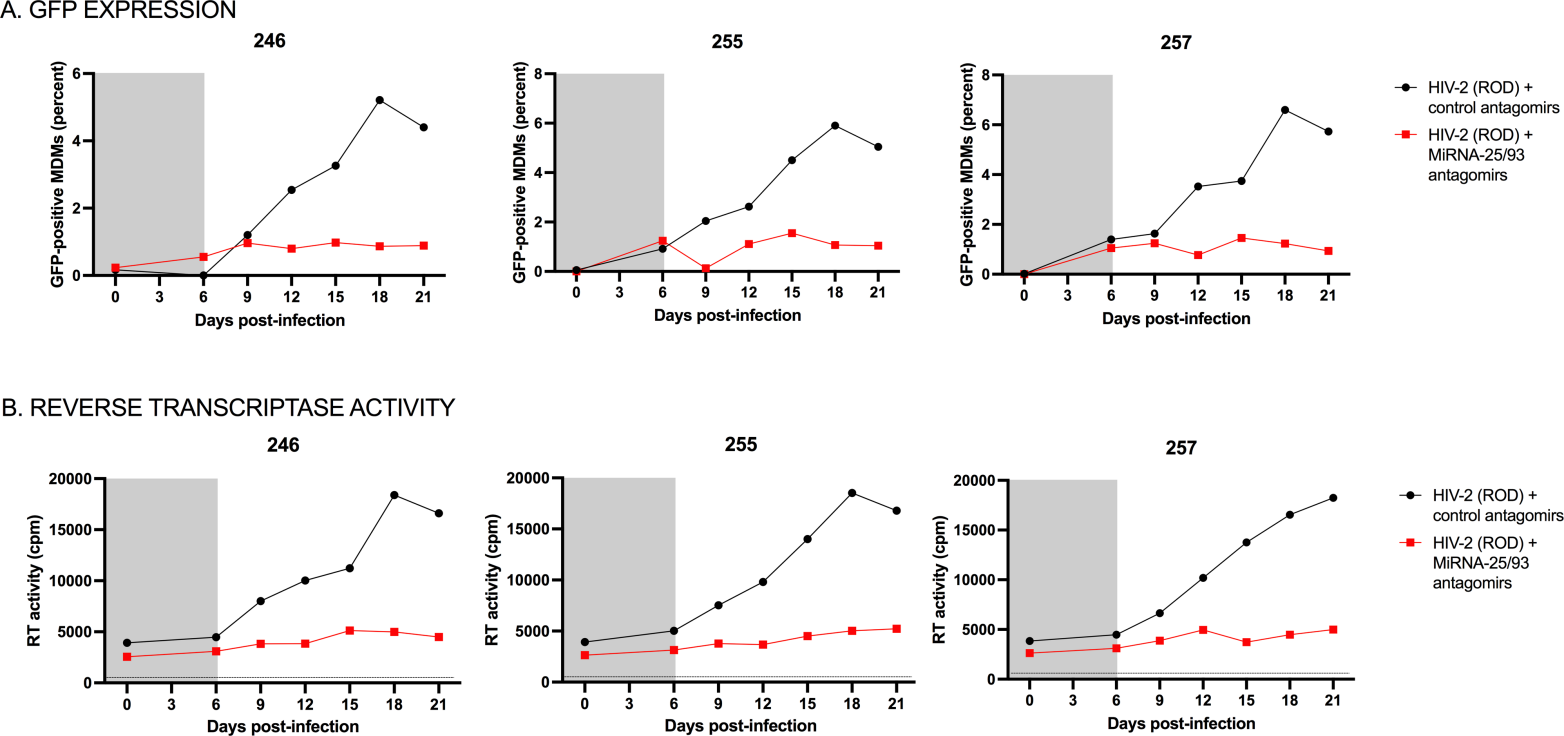
MicroRNA 25 and 93 antagonists reduce HIV-2 spread and kinetics of virus production in human macrophages. MDMs were transfected with control or a mix of microRNA 25 and 93 antagomirs after 6 days (depicted in gray) of GFP-expressing HIV-2 (ROD) infection. (**A**) Viral spread in the cell culture as per the frequency of viral infection (% GFP-expressing macrophages) at each time point. (**B**) Virus production as assessed by the RT activity in virus pellets recovered from cell supernatants at the indicated time points.

The enhanced expression of microRNAs 25 and 93 in HIV-1-infected macrophages, and the ensuing reduction of *MARCH1* mRNAs, leads to less MARCH1 protein and thus greater viral incorporation of Env glycoproteins, resulting in the release of fully infectious viral particles and an optimal viral spread in the cell culture, as demonstrated for HIV-1 (14). Indeed, the addition of a mix of microRNA-25/93 antagonists (antagomirs) to MDMs reduces the spread of HIV-1 infection (14). We tested if this was also the case for HIV-2 (ROD) (Fig. 2). In order to compensate for the much lower HIV-2 infection rates (as compared to HIV-1; Fig. S1) and to allow for thorough HIV-2 infection prior to the addition of the antagomirs, infected macrophage cultures were transfected with microRNA-25/93 antagomirs, following 6 days of infection. As shown in Fig. 2, treatment of MDMs with microRNA-25/93 antagomirs reduced both the levels of GFP+ cells (as a marker of macrophage infection) and the levels of virus production as measured by viral reverse transcriptase (RT) activity in pelleted viral particles from infected MDM cultures derived from three distinct donors. This suggests that the modulation of *MARCH1* mRNA by microRNAs 25 and 93 impacts the efficiency of the HIV-2 spread of infection.

**Fig 3:**
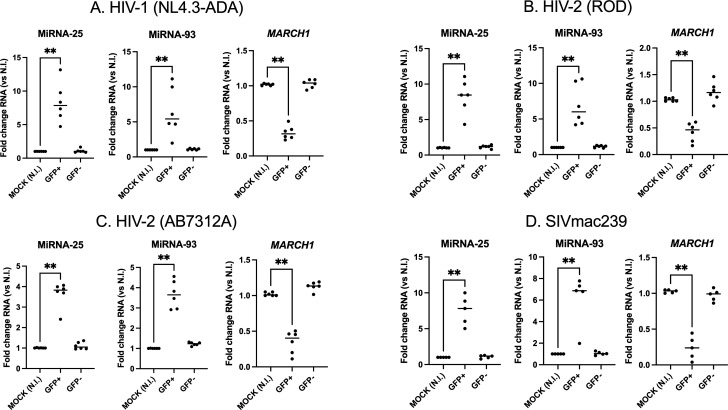
HIV-1, HIV-2, and SIVmac239 enhance microRNAs 25 and 93 expression and downregulate *MARCH1* mRNA in infected differentiated THP-1-CD4-CCR5 cells. Cells were infected for 36 h with the indicated VSV-G pseudotyped GFP-expressing viruses and sorted, as described in Materials and Methods. Total RNAs of the GFP+/GFP− cells were then extracted, reverse-transcribed, and microRNAs 25 and 93, as well as *MARCH1* mRNA, quantified by qRT-PCR. Fold changes are compared to uninfected cells (Mock, NI). (**A**) HIV-1 (NL4.3-ADA). (**B**) HIV-2 (ROD). (**C**) HIV-2 (AB7312A). (**D**) SIVmac239. **P < 0.01, using the Mann-Whitney U test.

Given the much lower virus infection rate obtained with SIVmac239 and HIV-2 compared to HIV-1 (Fig. S1), and to conduct mechanistic studies, we generated a CD4- and CCR5-expressing (Fig. S2) human monocyte THP-1 cell line, which, upon differentiation into macrophage-like cells, can be efficiently infected by either HIV-1, HIV-2, or SIVmac (Fig. S3). As shown in Fig. 3, microRNAs 25 and 93 were increased in the GFP+ sorted population of differentiated THP1-CD4-CCR5 cells following infection of either HIV-1 NL4.3-ADA, HIV-2 (ROD or AB7312A strains), or SIVmac239, while the levels of *MARCH1* mRNA were reduced to the same extent in these cells as in primary macrophages, thus validating this cellular model (Fig. 3).

### β-catenin and β-TrCP are critical for the increase of microRNAs 25 and 93 in HIV-2 or SIVmac-infected differentiated THP-1 cells

**Fig 4:**
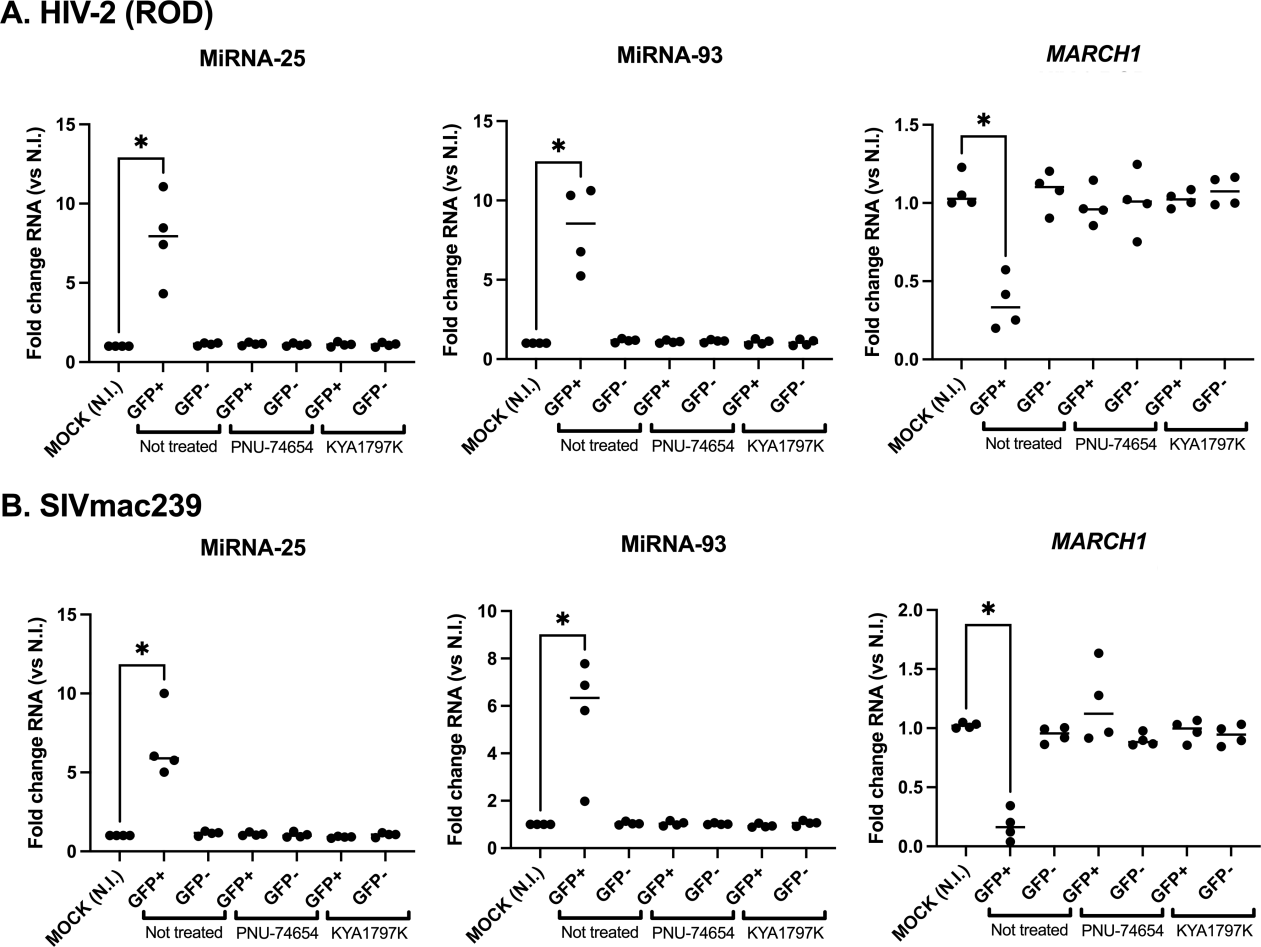
Role of β-catenin in the induction of microRNAs 25 and 93 in THP1-CD4-CCR5 cells infected with either HIV-2 ROD (**A**) or SIVmac239 (**B**). Cells were treated with the indicated β-catenin inhibitor as described in Materials and Methods, infected for 36 h, sorted for GFP expression, and their extracted RNAs processed for determining microRNAs 25 and 93, as well as *MARCH1* mRNA, expression by qRT-PCR. Values were then normalized to those of uninfected cells (NI, “mock”). *P < 0.05, using the Mann-Whitney U test.

We then investigated if the upregulation of microRNAs 25 and 93 in HIV-2 or SIVmac239-infected cells also involves β-catenin-driven transcription of these microRNAs. Differentiated THP1-CD4-CCR5 cells were pre-treated with either the β-catenin inhibitor PNU-74654, which inhibits the binding of β-catenin to the TCF4 transcription factor, or the β-catenin inhibitor KYA1797K, which accelerates the degradation of β-catenin; or control vehicles. Treated cells were then infected, in the presence of the inhibitors, with VSV-G pseudotyped particles of either HIV-2 ROD or SIVmac239 (Fig. 4) for 36 h, and sorted for GFP expression. In extension to previous data for HIV-1 (14), both the increases in microRNAs 25 and 93, as well as the decrease of *MARCH1* mRNA, were strongly inhibited by either PNU-74654 or KYA1797K in HIV-2 and SIVmac infected GFP+ cells (Fig. 4). These data suggest that the upregulation of microRNAs 25 and 93 expression in either HIV-2 or SIVmac239 infected GFP+ cells is β-catenin dependent.

**Fig 5:**
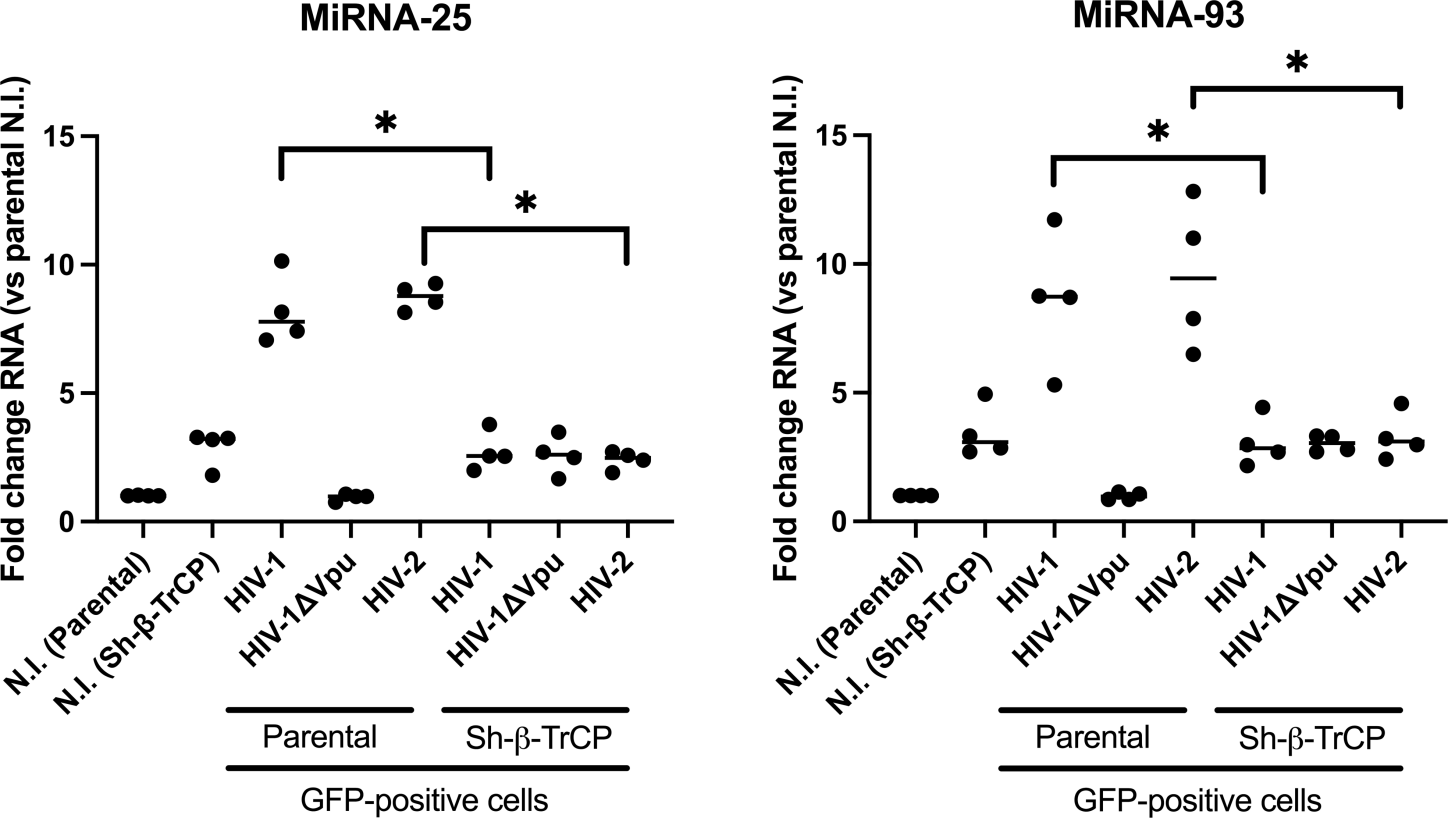
The β-TrCP substrate receptor of the SCF E3 ubiquitin ligase is involved in the induction of microRNAs 25 and 93 in HIV-2-infected cells. THP-1-CD4 cells (parental cell line) or THP-1-CD4-sh-β-TrCP cells, in which both β-TrCP1 and β-TrCP2 expression are silenced, were infected with the indicated VSV-pseudotyped viruses for 36 h as described in Materials and Methods. RNAs of sorted GFP-positive cells were then processed for microRNAs 25 and 93 expression by qRT-PCR. Values obtained were normalized to those of the uninfected THP-1-CD4 parental cells (NI, “Parental”). *P < 0.05, using the Mann-Whitney U test.

Through its interaction with β-TrCP, HIV-1 Vpu stabilizes β-catenin in the infected cell, which leads to the upregulation of microRNAs 25 and 93 (14). Given that HIV-2 does not encode for Vpu, but upregulates microRNAs 25 and 93 expression by a β-catenin-dependent mechanism, we investigated whether β-TrCP also plays a role in this process. Using shRNA-encoding lentiviruses, we generated a THP1-CD4 cell line knocked down for the expression of β-TrCP1 and β-TrCP2. The silencing of β-TrCP1 and β-TrCP2 mRNA expression was confirmed in the new THP1-CD4-sh-β-TrCP cell line obtained following clonal selection (Fig. S4A). Furthermore, HIV-1-infected THP1-CD4-sh-β-TrCP cells did not downregulate surface BST2 (Fig. S4B, compare dot plots j and k), which is usually reduced from the cell surface through a Vpu/β-TrCP-related mechanism following HIV-1 infection (Fig. S4B, compare dot plots d and e) (17, 18). To determine if β-TrCP is involved in the upregulation of microRNAs 25 and 93, differentiated parental THP-CD4 or THP1-CD4-sh-β-TrCP cells were infected with VSV-pseudotyped HIV-1 (NL4.3-ADA), Vpu-deficient NL4.3-ADA HIV-1, or HIV-2 (ROD) and sorted for GFP expression. The levels of microRNAs 25 and 93 were then determined in the GFP+ sorted cells (Fig. 5). Interestingly, non-infected THP1-CD4-sh-β-TrCP cells exhibited a threefold increase in basal expression of microRNAs 25 and 93 (Fig. 5) as compared to their parental THP-1-CD4 cells, likely due to the knockdown of the negative regulation of β-catenin exerted by β-TrCP. However, WT HIV-1 or WT HIV-2-infected GFP+ THP1-CD4-sh-β-TrCP cells did not upregulate microRNAs 25 and 93 (as compared to the non-infected controls) as did the parental THP1-CD4 cells (Fig. 5), suggesting that β-TrCP is involved in the upregulation of these microRNAs in HIV-1 and HIV-2-infected cells.

### The HIV-2 accessory proteins are not involved in microRNAs 25 and 93 upregulation in infected differentiated THP-1-CD4-CCR5 cells

**Fig 6:**
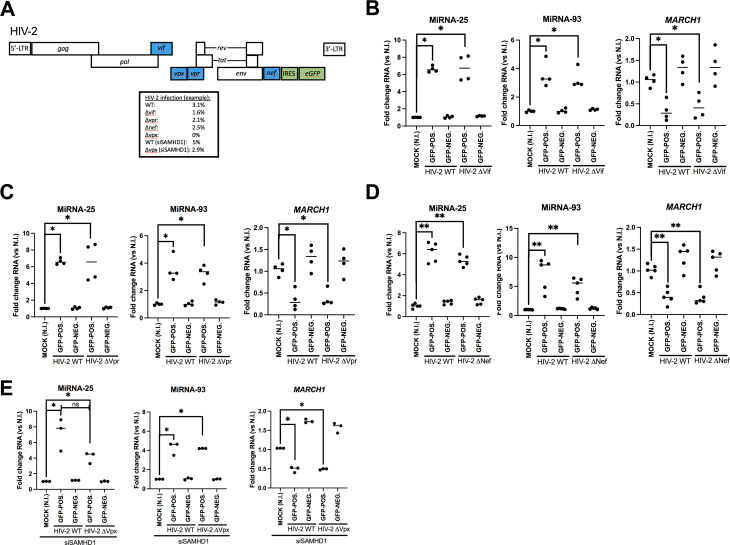
HIV-2/SIVmac accessory proteins do not induce microRNAs 25 and 93 in infected cells. (**A to E**) Differentiated THP1-CD4-CCR5 cells were infected with either the WT or accessory gene mutant HIV-2 AB7312A viruses and sorted based on GFP expression. Total RNAs were isolated and qRT-PCRs performed to measure microRNAs 25 and 93, and *MARCH1* mRNA, respectively. Values were normalized to those of uninfected cells (NI, “mock”). *P < 0.05, **P < 0.01, ns: not significant using the Mann-Whitney U test. (**A**) Scheme of the HIV-2 AB7312A-based provirus. Viruses deficient in one of the accessory genes in blue were used in panels B to E. Representative percentages of viral infection obtained (GFP+) for the WT or each mutant HIV-2 virus are shown. (**B**) Vif-deficient viruses. (**C**) Vpr-deficient viruses. (**D**) Nef-deficient viruses. (**E**) Prior to infection with WT or Vpx-deficient HIV-2 viruses, differentiated THP1-CD4-CCR5 cells were treated with siSAMHD1 for 48 h, and then processed similarly to the cells infected with the other accessory protein mutant viruses.

Given that the HIV-1 accessory protein Vpu drives the upregulation of microRNAs 25 and 93 in HIV-1-infected cells, but is not encoded by HIV-2 or SIVmac, we set out to determine if one of the HIV-2 accessory proteins is involved in this process. A panel of constructs derived from the HIV-2 strain AB7312A that are defective in the expression of either accessory proteins Vif, Vpr, Nef, or Vpx (Fig. 6A) was used to test for the upregulation of microRNAs 25 and 93 in differentiated THP-1-CD4-CCR5 cells. As observed with the WT HIV-2 virus, all accessory gene-defective viruses enhanced microRNAs 25 and 93 and reduced *MARCH1* mRNA in the GFP+ infected cells (Fig. 6B through E), suggesting that neither HIV-2 Vif, Vpr, Nef, nor Vpx is involved in upregulation of microRNAs 25 and 93. It is important to note that in order to assess the effect of Vpx (Fig. 6E), cells were first knocked down for SAMHD1, a restriction factor antagonized by Vpx (34), using siRNAs specific for SAMHD1 (siSAMHD1) (Fig. S5). Indeed, Vpx-defective virus infection was not detectable in differentiated THP-1-CD4-CCR5 cells without the use of siSAMHD1 (Fig. 6A).

### HIV-2 glycoprotein expression increases the levels of microRNAs 25 and 93 in differentiated THP-1 cells

**Fig 7:**
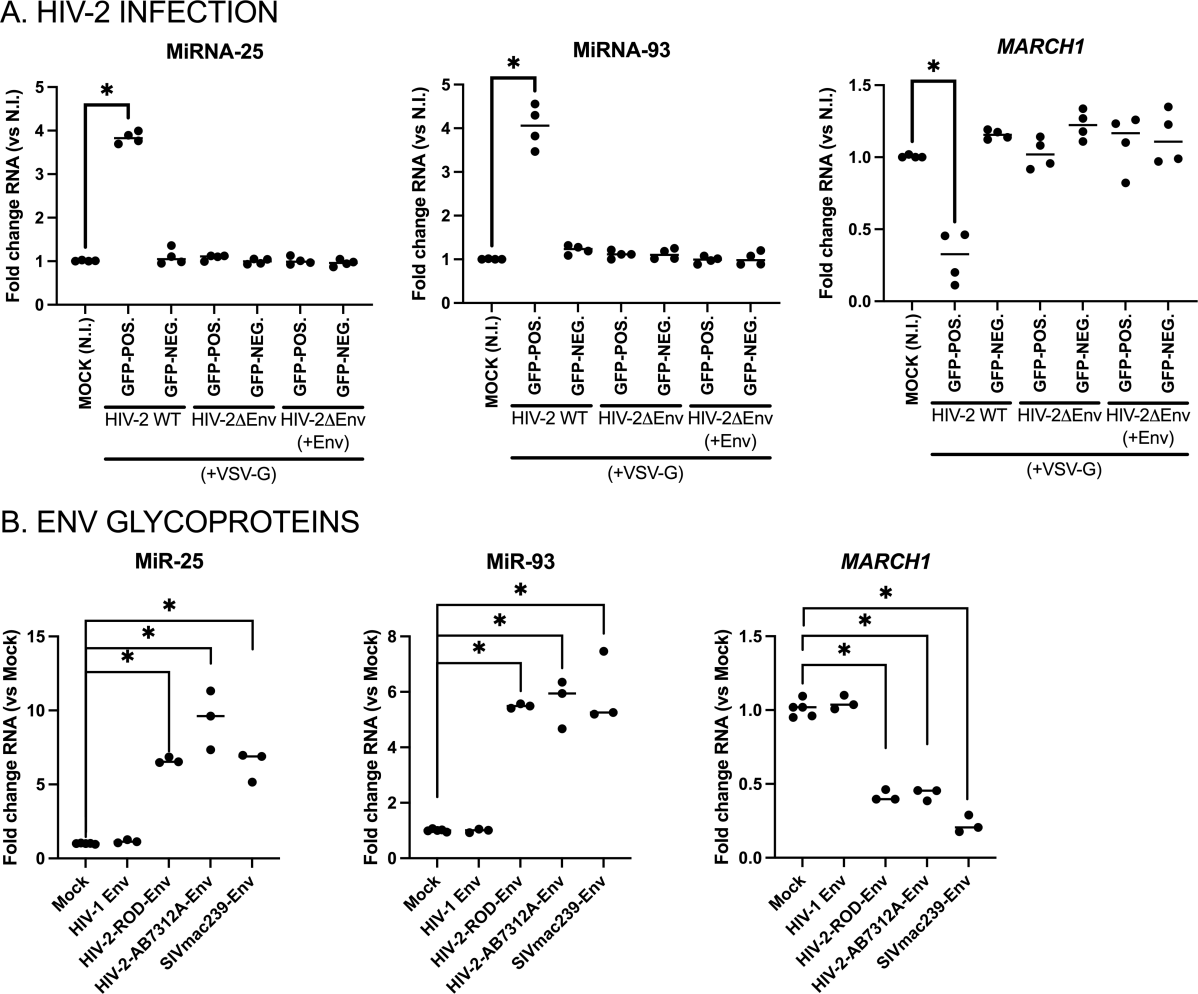
HIV-2 Env glycoproteins induce microRNAs 25 and 93 expression, leading to a reduction of *MARCH1* mRNA in macrophage-like THP-1 cells. (**A**) Differentiated THP-1-CD4-CCR5 cells were infected with the specified VSV-G pseudotyped, GFP-expressing HIV-2 (AB7312A-derived) viruses for 36 h and sorted into GFP+/GFP− populations, and their total RNAs were extracted. The levels of microRNAs 25 and 93, as well as *MARCH1* mRNA, were then determined by qRT-PCR and normalized to those of uninfected cells (NI, “mock”). (**B**) Differentiated THP-1 cells were transfected with the indicated lentiviral Env-expressing DNA constructs, which also encode for GFP. GFP-expressing cells were then sorted, and their total RNAs were extracted. The levels of microRNAs 25 and 93, as well as *MARCH1* mRNA, were then determined by qRT-PCR and normalized to mock-transfected cells. *P < 0.05, using the Mann-Whitney U test.

The HIV-2 Env glycoproteins have been shown to exhibit Vpu-like activity in regards to the enhancement of virus release by antagonizing host restriction factor BST2 (35). We therefore investigated if HIV-2 glycoproteins have an effect on the expression levels of microRNAs 25 and 93 in infected differentiated THP-1-CD4-CCR5 cells. For this, we produced WT or *env*-defective HIV-2 (AB7312A-based), or *env*-defective HIV-2 having incorporated HIV-2 Env (AB7312A) glycoproteins in *trans*, that were all VSV-G-pseudotyped (Fig. 7). We also ensured that VSV-G pseudotyping did not hinder the incorporation of HIV-2 glycoproteins in *trans* (Fig. S6A). Differentiated THP-1-CD4-CCR5 cells infected with each of these HIV-2 viruses were then sorted based on the presence of GFP, and the expression of microRNAs 25 and 93, and that of *MARCH1* mRNA was determined for each case. The *env*-defective HIV-2 lost the ability to upregulate microRNAs 25 and 93 expression and to reduce *MARCH1* mRNA, as compared to the WT HIV-2 (AB7312A) (Fig. 7A). Differentiated THP-1-CD4-CCR5 cells did not upregulate microRNAs 25 and 93 nor reduce *MARCH1* expression (Fig. 7A) when exposed to *env*-defective HIV-2 particles pseudotyped with HIV-2 Env (Fig. S6A). Taken together, these results suggest that active synthesis of HIV-2 Env glycoproteins in infected cells is necessary for microRNAs 25 and 93 upregulation.

In order to validate the impact of HIV-2 glycoproteins on microRNAs 25 and 93 expression, we directly transfected selected HIV-1, HIV-2, or SIVmac239 Env glycoprotein-encoding DNA constructs into THP-1 monocytes which were then differentiated for 48 h (Fig. 7B). These plasmids also include a *GFP* reporter gene, which enabled efficient sorting of transfected cells and obtaining enriched total RNA samples for microRNA expression analyses. Detection of a carboxy-terminal AU1 epitope tag on the Env glycoproteins suggested their specific expression in the GFP-positive cells, as shown using confocal microscopy (Fig. S6B). Importantly, sorted GFP-positive differentiated THP-1 cells expressing HIV-2 (ROD), HIV-2 (AB7312A), or SIVmac239 glycoproteins significantly enhanced their levels of microRNAs 25 and 93, while reducing *MARCH1* mRNA (Fig. 7B), as compared to mock-transfected cells. This was not the case for differentiated THP-1 cells transfected with HIV-1 (NL4.3) Env glycoproteins, suggesting that the upregulation of microRNAs 25 and 93 resulted from the ectopic expression of HIV-2/SIVmac glycoproteins. These results indicate that HIV-2/SIVmac Env glycoprotein expression is sufficient to induce the upregulation of microRNAs 25 and 93.  

## DISCUSSION

One important function of HIV accessory proteins is to counter host antiviral factors that restrict viral replication (15, 16). Recently, we have shown that HIV-1 Vpu counters the restriction exerted by MARCH1 on Env incorporation and viral infectivity by enhancing microRNAs 25 and 93, which target *MARCH1* mRNAs in infected macrophages (14). Remarkably, we also observed upregulation of these anti-MARCH1 microRNAs in HIV-2 and SIVmac239-infected cells (14). Given that both HIV-2 and SIVmac239 do not encode for a *vpu* gene, it was compelling to identify what viral factor was enhancing the levels of these two microRNAs in infected macrophages and to define the host pathways involved in the process.

We here show that microRNAs 25 and 93 are upregulated in infected monocyte-derived macrophages in the case of two different strains of HIV-2 (the HIV-2 ROD-derived ROD10 and the primary recombinant isolate AB7312A; Fig. 1; Fig. S1), and SIVmac239, suggesting that this is a common feature occurring upon primate lentivirus infection. The strong reduction of HIV-2 replication observed when MDMs are pretreated with inhibitors of these microRNAs further established their importance in HIV-2 replication as MARCH1 restriction antagonists (Fig. 2). Given that HIV-2 infection in MDMs is inefficient (36), limiting mechanistic studies, we modified THP-1-CD4 cells (37, 38) to additionally express higher levels of CCR5 (Fig. S2), enabling efficient second round infection. The differentiated THP-1-CD4-CCR5 cells had a greater affinity to HIV-2 infection as compared to MDMs (Fig. S1 and S3). Infected cells upregulated microRNAs 25 and 93 and reduced *MARCH1* mRNA following either HIV-1, HIV-2, or SIVmac239 infection, thus validating this cell model to study the interplay between the levels of microRNAs 25 and 93 and *MARCH1* mRNA expression (Fig. 3).

Interestingly, β-catenin inhibitors prevented the upregulation of microRNAs 25 and 93 in either HIV-2- or SIVmac239-infected THP-1-CD4-CCR5 cells (Fig. 4), as was the case for HIV-1, which we had previously shown to be Vpu-dependent (14). This suggests that a common β-catenin-dependent mechanism is used by HIV-1, HIV-2, and SIVmac239 for driving the upregulation of the two anti-MARCH1 microRNAs in infected macrophages. In THP-1-CD4 cells expressing shRNAs for β-TrCP1/2 (THP-1-CD4-sh-β-TrCP, Fig. S4), no increase of microRNAs 25 and 93 was observed following either HIV-1 or HIV-2 infection (Fig. 5), suggesting that the SCF-β-TrCP E3 ubiquitin ligase complex is involved in the upregulation of these two anti-MARCH1 microRNAs. In HIV-1-infected cells, Vpu downregulates CD4 (22) and BST2 (23) by linking these proteins to the SCF complex and increases cellular β-catenin by displacing β-TrCP toward other targets (14, 26), resulting in the microRNAs 25 and 93 upregulation. The fact that HIV-2 also appears to modulate these microRNAs through a β-TrCP and β-catenin-dependent mechanism suggests a convergence in the host pathways targeted by both HIV-1 and HIV-2, yet using a different mechanism, given the absence of Vpu in the case of HIV-2.

Given the role of HIV accessory proteins in the viral response to host antiviral factors (15, 16), we tested if they had any function in the upregulation of microRNAs 25 and 93. Having ruled out any involvement of HIV-2 Vif, Vpr, Vpx, or Nef accessory proteins in the modulation of these two microRNAs (Fig. 6), we focused on the possible effect of other HIV-2 proteins. HIV-2 glycoproteins have been shown to harbor Vpu-like functions, such as counteracting BST2 viral tethering activity (39). Indeed, HIV-2 downregulates BST2 from the cell surface, as we observed in HIV-2-infected THP-1-CD4 cells (Fig. S4B). In the case of SIV, the accessory protein Nef has mainly retained anti-BST2 activity (40, 41), although some SIV glycoproteins have evolved to have this function (42). Given their Vpu-like function, it was compelling to test if HIV-2 Env was involved in microRNAs 25 and 93 regulation. Remarkably, neither infection with Env-defective VSV-G-pseudotyped HIV-2, nor HIV-2Δ*Env* viruses pseudotyped with HIV-2 glycoproteins (Fig. S6A) enhanced the two microRNAs expression in infected cells, suggesting that HIV-2 Env biosynthesis was necessary for their upregulation (Fig. 7A). This finding was validated by transient expression of HIV-2 Env of epidemic group A (ROD) and group AB (AB7312A) (Fig. 7B) which strongly induced the expression of the two microRNAs; this also suggests that both epidemic HIV-2 groups A and B share this property (Fig. 7B; Fig. S6B), given that Env of AB7312A clusters closely to HIV-2 group B (43). Interestingly, while HIV-1 Env expression had no effect on the expression of microRNAs 25 and 93 (Fig. 7B; Fig. S6B), SIVmac239 glycoproteins did upregulate the anti-MARCH1 microRNAs (Fig. 7B). These combined observations suggest that HIV-2 glycoproteins induce microRNAs 25 and 93 by a process that involves β-catenin and β-TrCP. However, careful analysis of the cytoplasmic tail of HIV-2 glycoproteins did not reveal any known β-TrCP interacting di-serine containing consensus sequence (22), as found, for example, within HIV-1 Vpu. These data also suggest that HIV-2 glycoproteins counter both the MARCH1 and BST2 (35, 39) viral restriction factors affecting virus infectivity and release in myeloid cells, in cooperation with early acting viral proteins, such as Nef, that antagonize SERINC5 restriction on HIV-2 (44) and downmodulate CD4 for effective release of fully infectious virions (45). Such a sequential effect via viral proteins expressed at early and late stages of infection is likely to ensure optimal viral production and spread.

The constitutive, natural ubiquitination of BST2 is regulated by NEDD4 and MARCH8, and not by β-TrCP (46), which is consistent with the fact that HIV-2 glycoproteins do not use β-TrCP-mediated ubiquitination to counter BST2 antiviral activity, instead redistributing and sequestering BST2 into the Golgi compartment, without reducing its total cellular levels (35, 39). This is also the case for some SIV glycoproteins (42). We still observed a reduction of BST2 from the surface of HIV-2-infected THP-1-CD4-sh-β-TrCP cells (Fig. S4B). However, in this context, we did not observe an upregulation of the anti-MARCH1 microRNAs (Fig. 5), suggesting that β-TrCP is involved in this process. Thus, HIV-2 glycoproteins may sequester and interact with both β-TrCP and BST2 in a way that does not lead to BST2 ubiquitination. Indeed, BST2 binding by the HIV-2 Env ectodomain (47) would allow for such conditions. Remarkably, neither HIV-2 Env nor BST2 possesses a known β-TrCP-binding motif, and thus recruitment of β-TrCP to HIV-2 Env is likely to involve an intermediate binding partner. This would then lead to a β-catenin buildup and the enhanced expression of microRNAs 25 and 93.

Our finding that several lentiviruses have evolved different mechanisms, yet involving similar host factors, to enhance microRNAs 25 and 93 as a means to antagonize the myeloid-specific MARCH1 restriction on viral infectivity underlines the importance of this pathway in lentivirus replication in macrophages. MARCH1 reduces glycoprotein incorporation into viral particles (5, 14); in the case of HIV-1, MARCH1 redirects Env glycoproteins to a perinuclear region in infected macrophages (14). Interestingly, the Ebola virus glycoproteins are also sensitive to MARCH1 restriction (48) and display anti-BST2 activity (49), suggesting that these two mechanisms can coexist in the replication strategies of different viruses. However, whether other virus families that infect myeloid cells have developed any different countermeasures to MARCH1 antiviral activity remains to be determined.

## MATERIALS AND METHODS

### Plasmid constructs

Plasmids NL4.3-ADA-GFP-IRES-NEF, NL4.3-ADA-dU-GFP-IRES-NEF, described as HIV-1 (NL4.3-ADA) and HIVΔVpu in the text, and SV-CMV-VSV-G were previously described (14). SIVmac239-IRES-GFP (50, 51) was previously described, and HIV-2-ROD10-IRES-GFP (“ROD” in the text) was created using a similar cloning strategy. The proviral construct HIV-2 (AB7312A)-IRES-GFP, and its derived plasmids that do not encode for either Vpx, Vif, Vpr, or Nef accessory proteins, respectively, were previously described (33). The Env-negative HIV-2(AB7312A)-IRES-GFP was generated by site-directed mutagenesis. Lentiviral plasmids psPAX2 (a gift from Didier Trono, Addgene #12260) and SIV3+ (52) were previously described. Plasmids encoding AU1-tagged viral envelope glycoproteins from HIV-1 (NL4.3), SIVmac239, or HIV-2 (ROD10 or AB7312A) that include an IRES-GFP were also described elsewhere (53).

The pWPI-puro-CCR5 plasmid was generated by cloning the human CCR5 cDNA into the unique PmeI site of the pWPI plasmid and replacing the *GFP* gene following the internal ribosomal entry site (IRES) with a puromycin-resistance gene using the BmgBI and BstBI restriction enzyme sites.

The *GFP*-deleted versions pGIPZ-V2LHS-51187Δ*GFP* and pGIPZ-33325Δ*GFP* of plasmids pGIPZ-V2LHS-51187 and pGIPZ-33325, which express the shRNAs against β-TrCP2 (54) and against β-TrCP1 (55), respectively (see Table 1), were obtained by DNA blunting and ligation of the *GFP* flanking restriction enzyme sites BsrGI and BlpI.

**TABLE 1:** Primers used in this study (all are 5′ to 3′)

Primer name	Sequence
Mimics (Qiagen-Exiqon)	
MiRNA25-3p	CAUUGCACUUGUCUCGGUCUGA
MiRNA93-5p	CAAAGUGCUGUUCGUGCAGGUAG
Antagomirs (Qiagen-Exiqon)	
MiRNA25-3p	CAGACCGAGACAAGTGCAAT
MiRNA93-5p	TACCTGCACGAACAGCACTTT
For real-time qPCR of mRNAs	
hsMARCH1E1-258F1	TCCCAGGAGCCAGTCAAGGTT
hsMARCH1E2-385R1	CAAAGCGCAGTGTCCCAGTG
GAPDH-F	GCCATCAATGACCCCTTCAT
GAPDH-R	TTGACGGTGCCATGGAATTT
For real-time qPCR of microRNAs	
MiR-25-3p-loop	CAAGTGCAAGATATGTGAGACGTACGTTGAGTACGTCAAGTGAAGTTCAGACC
MiR-25-3pFWD	CAAGTGCAAGATATGTGAGACGTACGTTG
MiR-25-3pREV	GCATTGCACTTGTCTCGGTCTGA
MiR-93-5p-loop	GCACTTTGGCTAGCTATGCAGGTACAGTTGGTACCTGACTCTTGTTCTACCTGC
MiR-93-5pFWD	GCACTTTGGCTAGCTATGCAGGTAC
MiR-93-5pREV	ACAAAGTGCTGTTCGTGCAGGTAG
For shRNA lentivirus constructs	
β-TrCP1	CACATAAACTCGTATCTTA
β-TrCP2	CCAATTATCTGTTTGAAAT
For generating HIV-2(AB7312A)-IRES-GFP Env-negative construct	
FWD	GAGTAAGATGtgatgaAAGAATCTACTATTTGTTG
REV	CACTTGTCTGATGCAGAAGATG

### Chemicals and antibodies

The following reagents were used: β-catenin inhibitors KYA1797K (Sigma, #SML1831; 10 µM) and PNU-74654 (R&D Systems #3534/5; 200 µM); phorbol 12-myristate 13-acetate (PMA, Sigma #P8139; 100 nM). The antibodies used for flow cytometry, mouse anti-CD4-BV421 (#317433), rat anti-CCR5-PerCP-Cy5.5 (#359112), and mouse anti-BST2-PE (#348406) and their isotype controls were from Biolegend. Rabbit anti-AU1 tag (Bethyl/Fortis #A190-125A) was used for confocal microscopy, in combination with goat anti-rabbit Alexa647, or for western immunoblotting. Mouse anti-GAPDH FF26A/F9 (#649202) and rabbit anti-SAMHD1 (#12586-I-AP) were from Biolegend and Proteintech, respectively. Rabbit polyclonal anti-VSV was a gift of Laurent Poliquin (56).

### Cell culture and transfection, siRNA, and establishment of THP-1 cell lines

Isolation of MDMs and differentiation into macrophages was performed as previously described (14). MDMs or differentiated THP-1-CD4-CCR5 cells were transfected using Lipofectamine RNAi Max as previously described (14) with non-targeting controls or a mix of miRNA-25-3p (#YI04100613) and miRNA-93-5p (#YI04101031) antagomirs obtained from Qiagen (Exiqon), or siRNA for SAMHD1 from Dharmacon/GE Healthcare (siGENOME SMARTpool, #M-013950-00-0005). Non-targeting control RNAs were obtained from Dharmacon/GE Healthcare (siGENOME nontargeting 2, #D-001210-02-20) or Ambion/Thermo Fisher Scientific (#AM16104).

The THP-1 and THP-1-CD4 cell lines were previously described (37, 38). For all THP-1 cell lines, differentiated cells were obtained by treating cells with 100 nM PMA for 12 h and further incubation for 2 days without PMA, prior to use. The THP-1-CD4-CCR5 cell line was obtained following transduction of THP-1-CD4 cells with lentiviral vectors produced using pWPI-puro-CCR5 and selecting for puromycin-resistant THP-1-CD4-CCR5 cells.

The THP-1-CD4-sh-β-TrCP cell line was obtained by transducing THP-1-CD4 cells with a mix of lentiviruses generated using pGIPZ-V2LHS-51187Δ*GFP* and pGIPZ-33325Δ*GFP* and selection of puromycin-resistant cells.

THP-1 monocytes were transfected by DEAE-Dextran as described by Aneja et al. (57), differentiated, and GFP-positive cells sorted on a BD FACSAria III (BD Biosciences) cell sorter. Sorted cells were directly recovered in RLT lysis buffer (Qiagen) for total RNA extraction.

### Virus and lentiviral vector production, infection of MDMs and THP-1-CD4-CCR5 cells, and cell sorting of infected cells

The appropriate lentiviral vector plasmids (pGIPZ-V2LHS-51187Δ*GFP*, pGIPZ-33325Δ*GFP,* or pWPI-puro-CCR5) were transfected with psPAX2 and SV-CMV-VSV-G into HEK293T cells by calcium phosphate precipitation in order to generate the desired lentiviral vectors.

Viruses were produced by calcium phosphate transfection of proviral constructs in HEK293T cells and their titers determined as previously described (14), using the TZM-bl reporter cell line (58). To enhance the infection frequency, MDMs or differentiated THP-1 cells were pre-treated with VSV-G pseudotyped SIV3+ vectors (containing Vpx) and then infected with either NL4.3-ADA-GFP-IRES-NEF, NL4.3-ADA-dU-GFP-IRES-NEF, HIV-2-ROD10-IRES-GFP, HIV-2-AB7312A-IRES-GFP, or SIVmac239-IRES-GFP (all VSV-G pseudotyped), unless specifically stated differently, and at a multiplicity of infection of 1. After 36 h of infection, cells were harvested and sorted based on their GFP expression, using either a BD Influx (BD Biosciences) or a BD FACSAria Fusion (BD Biosciences) flow cytometer. Sorted cells were directly recovered in RLT lysis buffer (Qiagen) for total RNA extraction.

### RNA extraction, reverse transcription, and real-time qPCR analyses

Total cellular RNAs (including microRNAs) were extracted using RNeasy RNA extraction columns (Qiagen) according to the manufacturer’s instructions and stored at −80°C. Reverse transcription of RNAs was performed using SuperScript III reverse transcriptase (Invitrogen) with poly(dT); in the case of microRNAs, specific loop primers for the specific microRNAs were designed for two-tailed qRT-PCR (59) and added to the mix. For qRT-PCR, cDNAs and appropriate primers (Table 1) were added to SYBR green select master mix (Applied Biosystems) in 96-well plates and run on a ViiA96 thermocycler (ThermoFisher Scientific). *GAPDH* was used as a loading control, and ΔΔ*C_T_* variations were calculated and compared to those of uninfected cells.

### HIV-2 virus quantification by radioactive RT assay

HIV-2 released in culture supernatants was pelleted and measured by viral reverse transcriptase activity using a [H3]-deoxythymidine (dTTP) incorporation assay. Briefly, virus pellets were resuspended in 10 µL of 50 mM Tris-HCl, pH 7.5, 1 mM DTT, 20% glycerol, 0.25 mM KCl, and 0.25% Triton X-100 and frozen. Following thawing, 10 µL of 5× RT assay buffer (250 mM Tris-HCl pH 7.5, 37.5 mM MgCl_2_, 0.25% Triton X-100), 1.2 µL of 200 mM DTT, 5 µL (0.05 A_260_ units) of oligo-dT-poly-A (Roche), 0.1 μCu of [H3]-dTTP and 22.8 µL H_2_O were added to each sample, and kept at 37°C for 1 h. Samples were then placed on DE81 Whatman filters, washed in 2× SSC (300 mM NaCl, 30 mM sodium citrate, pH 7) and 95% ethanol, dried, and placed in vials with 7 mL CytoScint (MP biomedicals) and radioactivity measured on a scintillation counter.

### Flow cytometry

Differentiated THP-1 cells were collected by gentle scraping following a 10 min, 37°C incubation in phosphate-buffered saline (PBS)-EDTA (5 mM). Cells were then either fixed with 4% paraformaldehyde in PBS for GFP expression analyses or processed for flow immuno-cytometry. In this case, THP-1 cells were washed in FACS buffer (PBS containing 2% fetal bovine serum [FBS]) and Fc receptors blocked with a mix of human decomplemented plasma on ice for 1 h. Fluorochrome-labeled antibodies were added directly to the cells in the blocking solution, incubated for 1 h on ice, and the cells were washed three times in FACS buffer. Cells were then fixed with 4% paraformaldehyde in PBS, resuspended in PBS-EDTA, and analyzed on a BD Biosciences Fortessa cytometer equipped with the appropriate lasers. Detailed analyses were obtained using the FlowJo software package and https://floreada.io. Geometric mean fluorescence intensities were used.

### Sodium dodecyl sulfate-polyacrylamide gel electrophoresis and western immunoblot analyses

HIV-2-AB7312A-IRES-GFP Env-negative viruses, pseudotyped with either HIV-2-AB7312A, VSV-G, or both glycoproteins, were produced in HEK293T cells and pelleted. Differentiated THP-1-CD4-CCR5 cells were treated with siSAMHD1 or siControl and lysed as described previously (38). Viral or cellular protein lysates were separated by sodium dodecyl sulfate-polyacrylamide gel electrophoresis, and immunoblotting was performed as previously described (38).

### Confocal microscopy

Transfected non-differentiated THP-1 cells were transfected with plasmids expressing carboxy-terminally AU1-tagged HIV-1 glycoproteins, then differentiated and grown on coverslips, and then processed for confocal microscopy. Cells were washed with PBS, fixed with 4% paraformaldehyde in PBS at room temperature, permeabilized with 0.2% Triton X-100 for 10 min, and Fc receptors blocked with a mix of human decomplemented plasma on ice for 1 h. Following an hour incubation on ice with rabbit anti-AU1 epitope antibodies diluted 1:1,000 in blocking buffer, cells were washed three times in wash buffer (PBS containing 2% FBS) and then incubated with a mix of DAPI and goat anti-rabbit Alexa647 for 30 min on ice, washed three times, and mounted on slides. Cells were then imaged using a Zeiss 710 confocal microscope, with a 40×/1.3 oil PlanApo objective and the Zeiss Zen software package.

### Statistics

Statistical analyses were performed in GraphPad Prism 9 using the non-parametric Mann-Whitney rank test. Statistical significance is indicated in the figures (*, *P* < 0.05; **, *P* < 0.01).

## Data Availability

All data are within the article and supplemental material.

## SUPPLEMENTAL MATERIAL

Fig. S1 jvi.01663-25-s0001.pdf Representative flow cytometry data for GFP+/GFP- sorted MDMs from one blood donor.

Fig. S2 jvi.01663-25-s0002.pdf CD4 and CCR5 expression, as determined by flow cytometry, in undifferentiated and differentiated THP-1, THP1-CD4, and THP1-CD4-CCR5 cell lines.

Fig. S3 jvi.01663-25-s0003.pdf Infection of THP1-CD4-CCR5 cells.

Fig. S4 jvi.01663-25-s0004.pdf Characterization of THP-1-CD4-sh-β-TrCP cells.

Fig. S5 jvi.01663-25-s0005.pdf Validation of siSAMHD1.

Fig. S6 jvi.01663-25-s0006.pdf Detection of Env glycoproteins.

ASM does not own the copyrights to Supplemental Material that may be linked to, or accessed through, an article. The authors have granted ASM a non-exclusive, world-wide license to publish the Supplemental Material files. Please contact the corresponding author directly for reuse.
