# Supplementary material for: HIV-2 glycoproteins upregulate microRNAs 25 and 93 to counter the MARCH1 antiviral effect in macrophages
Source: J Virol. 2025 Nov 24;99(12):e01663-25. doi: 10.1128/jvi.01663-25 (PMC12724348; doi:10.1128/jvi.01663-25)
Supplement: Fig. S1 — Representative flow cytometry data for GFP+/GFP- sorted MDMs from one blood donor. [file jvi.01663-25-s0001.pdf]

Suppl. figure 1 (related to figure 1). Representative flow cytometry data for GFP+/GFP- sorted MDMs from one blood donor. Frequency of viral infection (% GFP+ cells) are shown for GFP-expressing HIV-1 NL4.3-ADA, HIV-2 ROD, HIV-2 AB7312A, and SIVmac239 at 36 hours post-infection.

INFECTION OF MONOCYTE-DERIVED MACROPHAGES

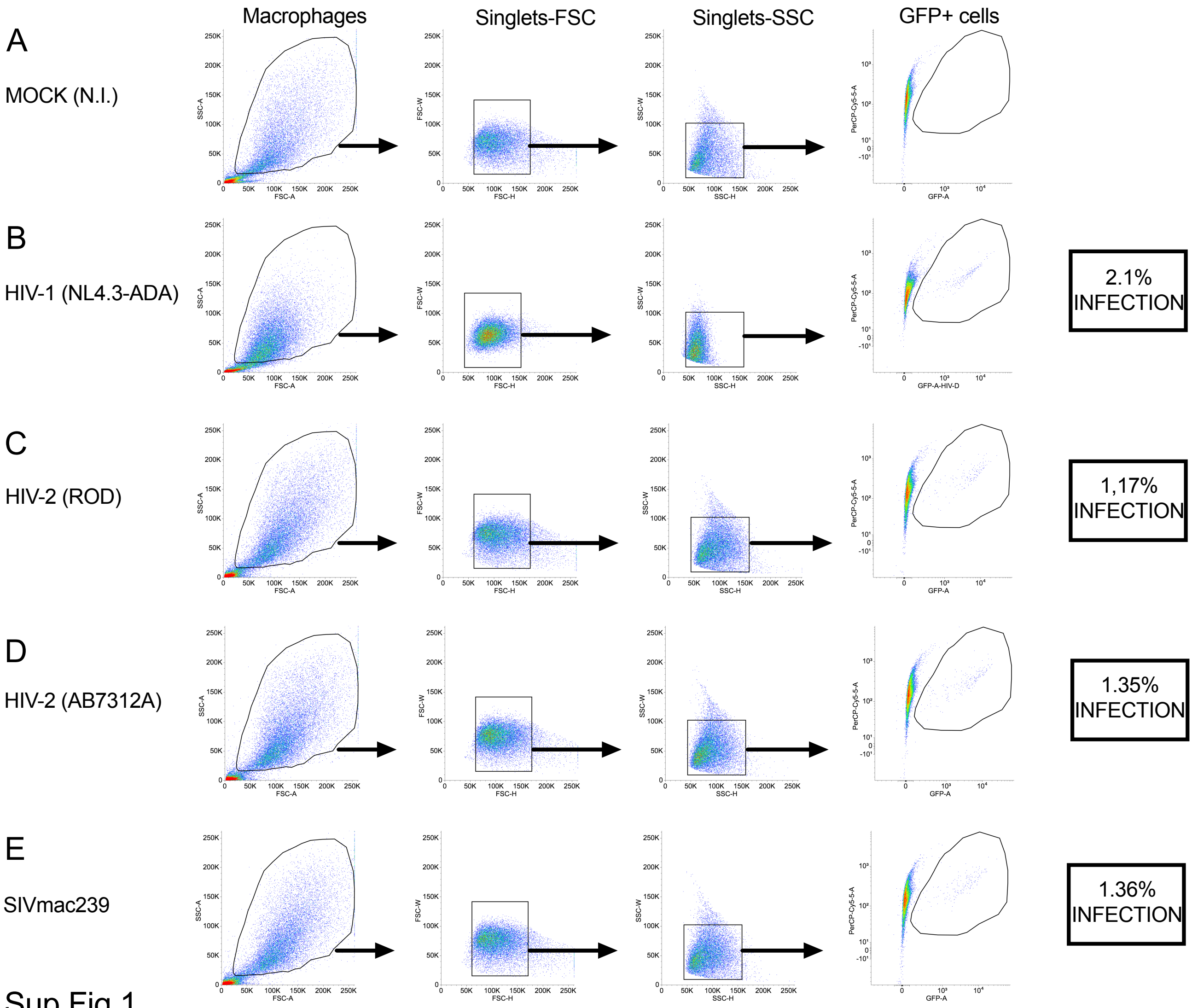

Sup Fig 1
