# Supplementary material for: HIV-2 glycoproteins upregulate microRNAs 25 and 93 to counter the MARCH1 antiviral effect in macrophages
Source: J Virol. 2025 Nov 24;99(12):e01663-25. doi: 10.1128/jvi.01663-25 (PMC12724348; doi:10.1128/jvi.01663-25)
Supplement: Fig. S2 — CD4 and CCR5 expression, as determined by flow cytometry, in undifferentiated and differentiated THP-1, THP1-CD4, and THP1-CD4-CCR5 cell lines. [file jvi.01663-25-s0002.pdf]

Suppl. figure 2 (related to figure 3). CD4 and CCR5 expression, as determined by flow cytometry, in undifferentiated and differentiated THP-1, THP1-CD4 and THP1-CD4-CCR5 cell lines. Mean fluorescent intensities (MFI) are shown for isotype controls (black) and CD4 or CCR5 (blue) for each sample. Note that CD4 expression is downregulated during differentiation of THP-1 cells, while CCR5 is slightly upregulated. Ectopic expression of CD4 or CD4 and CCR5 promotes efficient infection of differentiated THP-1 cells.

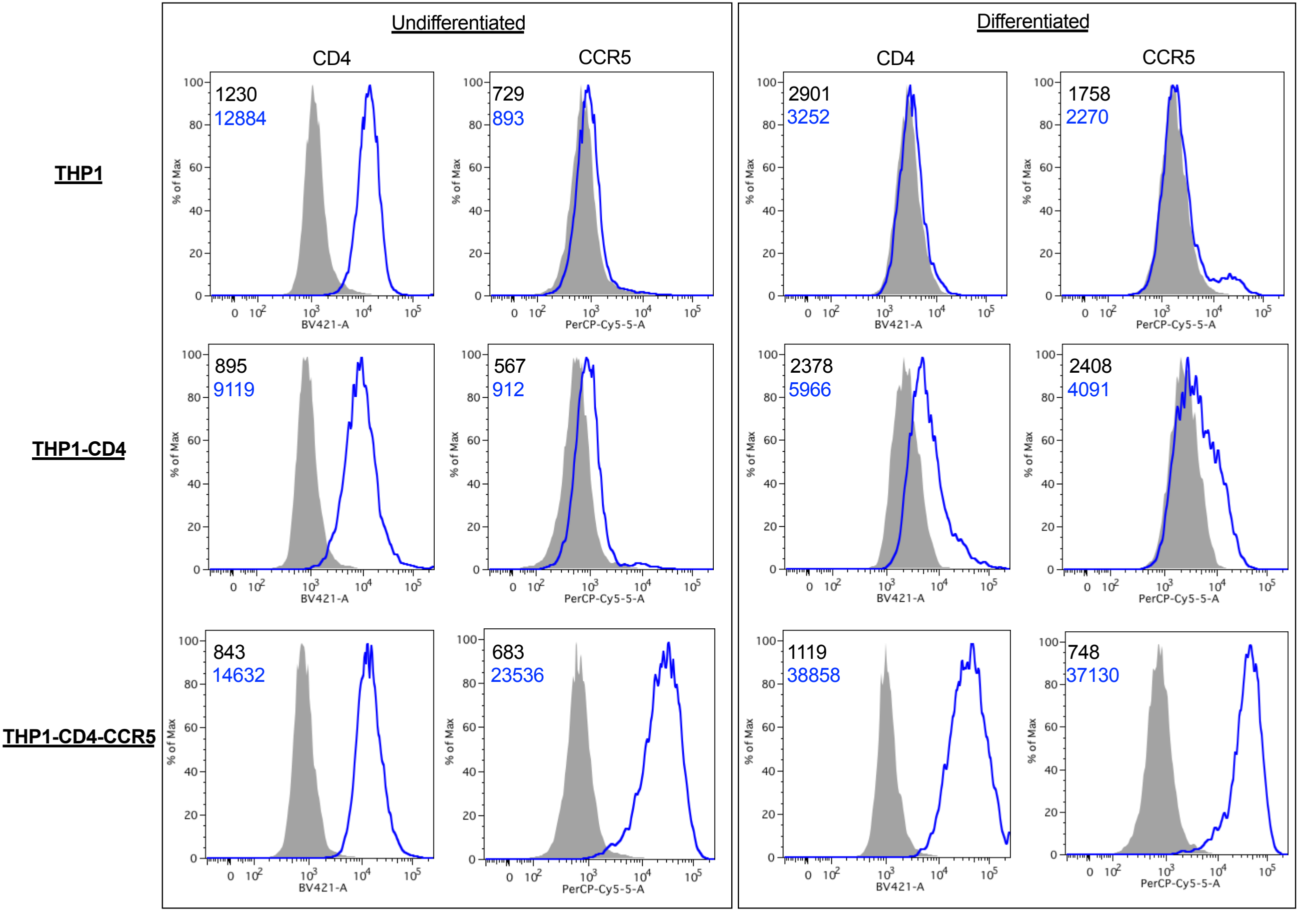

Undifferentiated
Differentiated
CD4
CCR5
CD4
CCR5
THP1
THP1-CD4
THP1-CD4-CCR5
1230
12884
729
893
2901
3252
1758
2270
895
9119
567
912
2378
5966
2408
4091
843
14632
683
23536
1119
38858
748
37130
% of Max
BV421-A
PerCP-Cy5-5-A

Sup Fig 2
