# Supplementary material for: HIV-2 glycoproteins upregulate microRNAs 25 and 93 to counter the MARCH1 antiviral effect in macrophages
Source: J Virol. 2025 Nov 24;99(12):e01663-25. doi: 10.1128/jvi.01663-25 (PMC12724348; doi:10.1128/jvi.01663-25)
Supplement: Fig. S3 — Infection of THP1-CD4-CCR5 cells. [file jvi.01663-25-s0003.pdf]

Suppl. figure 3 (related to figure 3). Infection of THP1-CD4-CCR5 cells. Representative flow cytometry data is shown for GFP+/GFP- sorted cells. Frequency of viral infection (% GFP+ cells) are shown for GFP-expressing HIV-1 NL4.3-ADA (B), HIV-2 ROD (C), HIV-2 AB7312A (D), and SIVmac239 (E) at 36 hrs post-infection. N.I.= not infected.

INFECTION OF DIFFERENTIATED THP-1-CD4-CCR5 CELLS

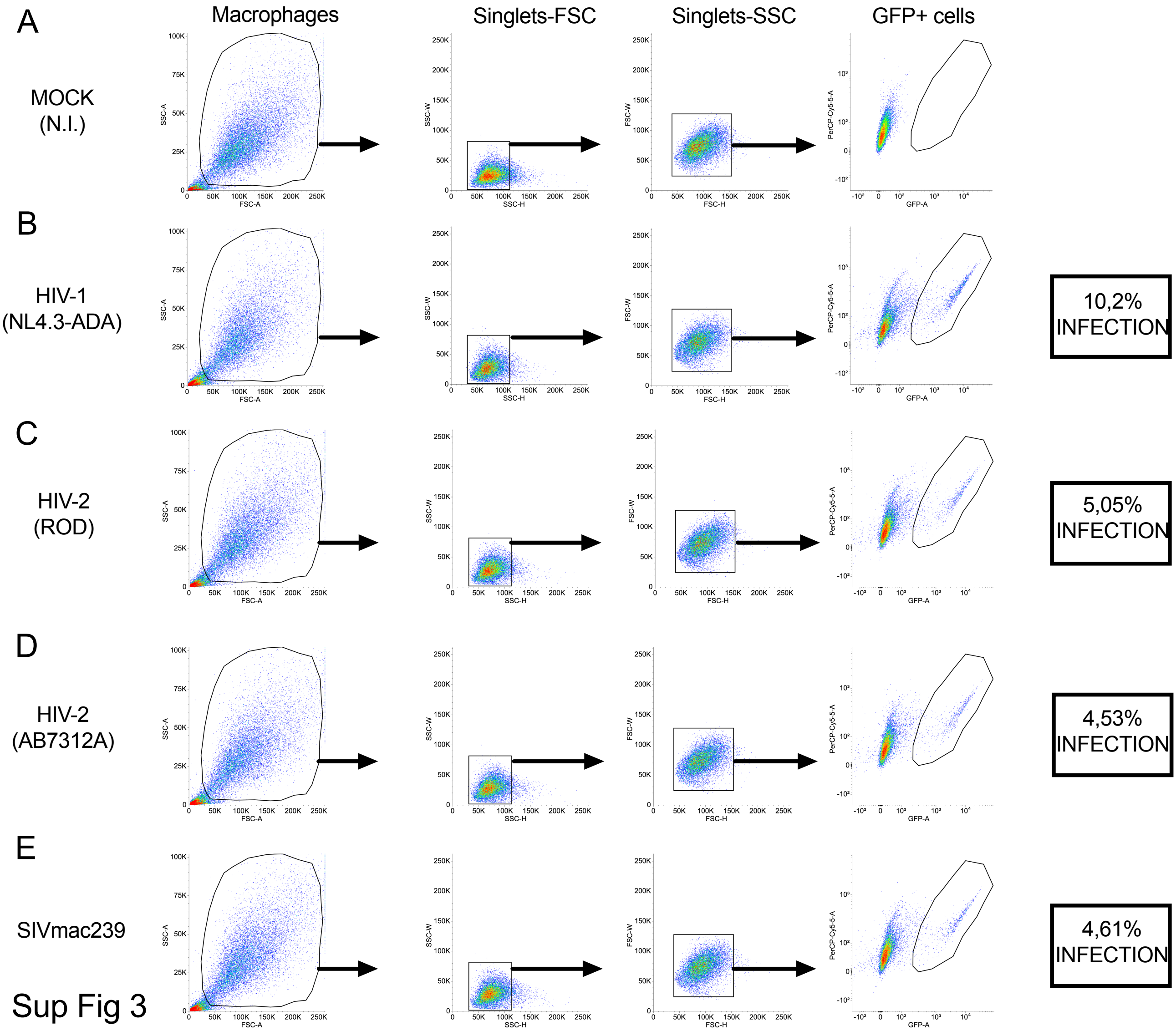

Sup Fig 3
