# Supplementary material for: HIV-2 glycoproteins upregulate microRNAs 25 and 93 to counter the MARCH1 antiviral effect in macrophages
Source: J Virol. 2025 Nov 24;99(12):e01663-25. doi: 10.1128/jvi.01663-25 (PMC12724348; doi:10.1128/jvi.01663-25)
Supplement: Fig. S4 — Characterization of THP-1-CD4-sh-β-TrCP cells. [file jvi.01663-25-s0004.pdf]

Suppl. figure 4 (related to figure 5). Characterization of THP-1-CD4-sh-β-TrCP cells. A. Real-time qPCR of β-TrCP 1 and 2 mRNAs in THP-1-CD4-sh-β-TrCP cells. Briefly, THP-1-CD4 cells were treated with a mix of lentiviral vectors encoding shRNAs targeting β-TrCP1 and β-TrCP2, and following cell culture, puromycin-resistant THP-1-CD4-sh-β-TrCP cells were obtained. Total RNAs of THP-1-CD4 and THP-1-CD4-sh-β-TrCP cells were extracted and tested by qRT-PCR for either β-TrCP1 or β-TrCP2 expression, and compared to the THP-1-CD4 parental line (set at 1). *$p < 0.05$, using the Mann-Whitney U test (n=3). B. Downregulation of surface BST2 is impaired in HIV-1 infected THP-1-CD4-sh-β-TrCP cells. THP-1-CD4 or THP-1-CD4-sh-β-TrCP cells were infected with the indicated VSV-pseudotyped viruses for 7 days, collected and stained with isotype control mAbs or anti-BST2. The levels of BST2 were then determined in the GFP-positive and GFP-negative cells by cytofluorometry. Shown is the mean fluorescent intensity (MFI) for the GFP-positive or GFP-negative cells. Note that the Vpu-mediated downregulation of surface BST2, which is dependent on β-TrCP, is impaired in THP-1-CD4-sh-β-TrCP cells as compared to the control, indicating that the β-TrCP function is impaired in THP-1-CD4-sh-β-TrCP cells.

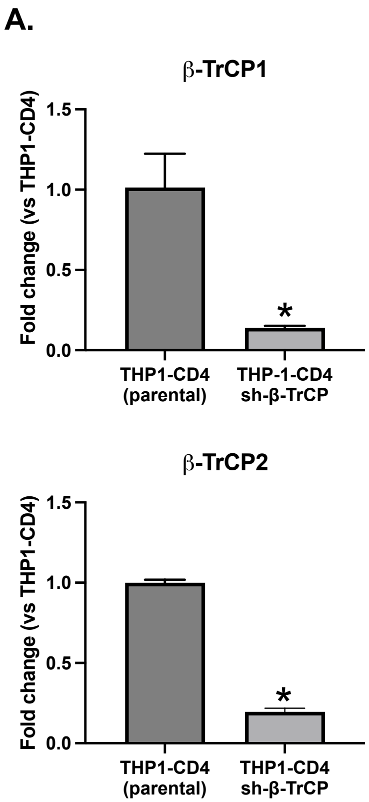
A.
β-TrCP1
Fold change (vs THP1-CD4)
1.5
1.0
0.5
0.0
*
THP1-CD4 (parental)
THP-1-CD4 sh-β-TrCP
β-TrCP2
Fold change (vs THP1-CD4)
1.5
1.0
0.5
0.0
*
THP1-CD4 (parental)
THP1-CD4 sh-β-TrCP

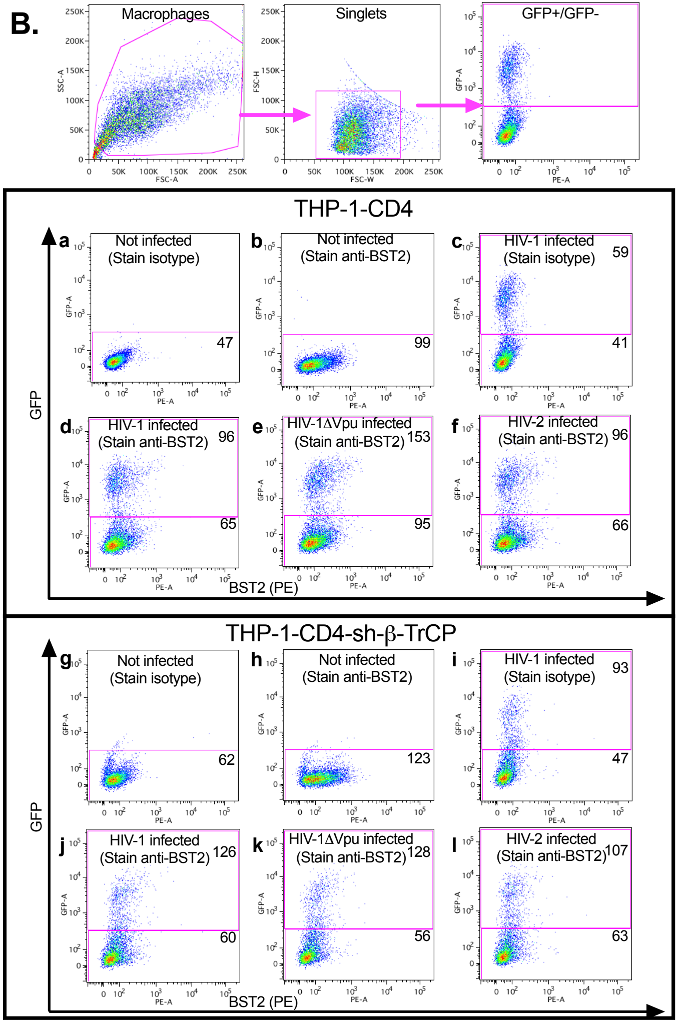
B.
Macrophages
Singlets
GFP+/GFP-
SSC-A
FSC-A
FSC-H
FSC-W
GFP-A
PE-A
THP-1-CD4
a Not infected (Stain isotype) 47
b Not infected (Stain anti-BST2) 99
c HIV-1 infected (Stain isotype) 59 41
d HIV-1 infected (Stain anti-BST2) 96 65
e HIV-1ΔVpu infected (Stain anti-BST2) 153 95
f HIV-2 infected (Stain anti-BST2) 96 66
GFP
BST2 (PE)
THP-1-CD4-sh-β-TrCP
g Not infected (Stain isotype) 62
h Not infected (Stain anti-BST2) 123
i HIV-1 infected (Stain isotype) 93 47
j HIV-1 infected (Stain anti-BST2) 126 60
k HIV-1ΔVpu infected (Stain anti-BST2) 128 56
l HIV-2 infected (Stain anti-BST2) 107 63
GFP
BST2 (PE)

Sup Fig 4
