# Supplementary material for: HIV-2 glycoproteins upregulate microRNAs 25 and 93 to counter the MARCH1 antiviral effect in macrophages
Source: J Virol. 2025 Nov 24;99(12):e01663-25. doi: 10.1128/jvi.01663-25 (PMC12724348; doi:10.1128/jvi.01663-25)
Supplement: Fig. S5 — Validation of siSAMHD1. [file jvi.01663-25-s0005.pdf]

Suppl. figure 5 (related to figure 6). Validation of siSAMHD1. A. Knockdown of SAMHD1 expression following siSAMHD1 treatment in differentiated THP-1-CD4-CCR5 cells. The cells were treated with siSAMHD1 or siControl as described in Materials and Methods for the indicated times, lysed and processed for Western blot analyses as described in Materials and Methods. The 48 hr siSAMHD1 treatment was consequently used for the infection of Vpx-deficient HIV-2 viruses in figure 6E. B. Densitometry analysis of the Western blot shown in A.

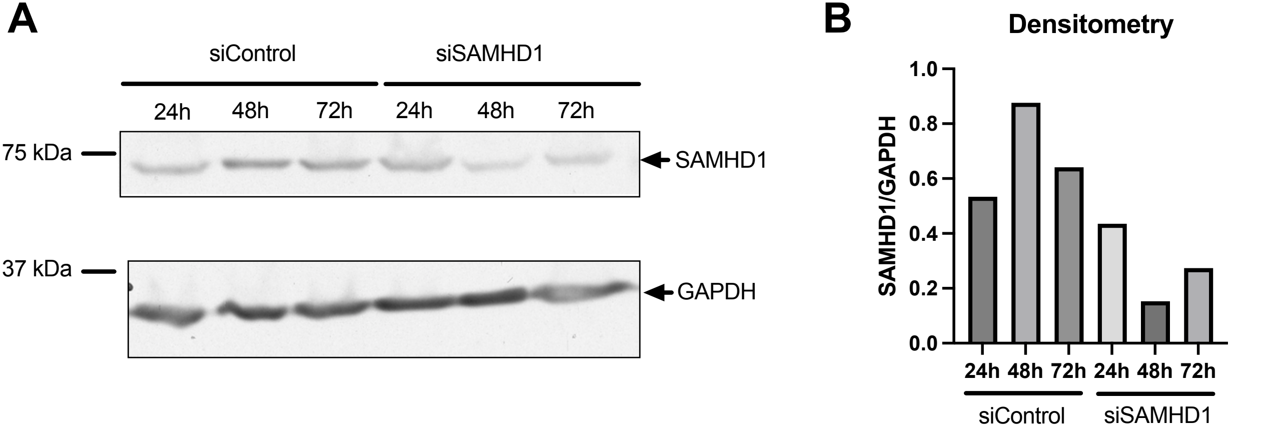
A
siControl
siSAMHD1
24h
48h
72h
24h
48h
72h
75 kDa
SAMHD1
37 kDa
GAPDH
B
Densitometry
SAMHD1/GAPDH
1.0
0.8
0.6
0.4
0.2
0.0
24h
48h
72h
24h
48h
72h
siControl
siSAMHD1

Sup Fig 5
