# Supplementary material for: HIV-2 glycoproteins upregulate microRNAs 25 and 93 to counter the MARCH1 antiviral effect in macrophages
Source: J Virol. 2025 Nov 24;99(12):e01663-25. doi: 10.1128/jvi.01663-25 (PMC12724348; doi:10.1128/jvi.01663-25)
Supplement: Fig. S6 — Detection of Env glycoproteins. [file jvi.01663-25-s0006.pdf]

Suppl. figure 6 (related to figure 7). Detection of Env glycoproteins. A. Incorporation of HIV-2 Env (transmembrane glycoprotein gp36) and VSV-G glycoproteins in HIV-2 viral particles used in figure 7 (A). Viruses were produced in HEK-293T cells using the appropriate constructs, pelleted and equal amounts of virus (based on reverse transcriptase activity) were processed for Western immunoblot analyses as described in Materials and Methods. Note that co-expression of VSV-G does not affect the levels of transmembrane Env glycoprotein incorporated into virions. B. Transfected THP-1 cells co-expressing GFP and the indicated Env glycoproteins. Env expression was detected by revealing the C-terminal AU1-tag present on the viral glycoproteins, as visualized by confocal microscopy. Representative examples are shown. Bar = 10 μm.

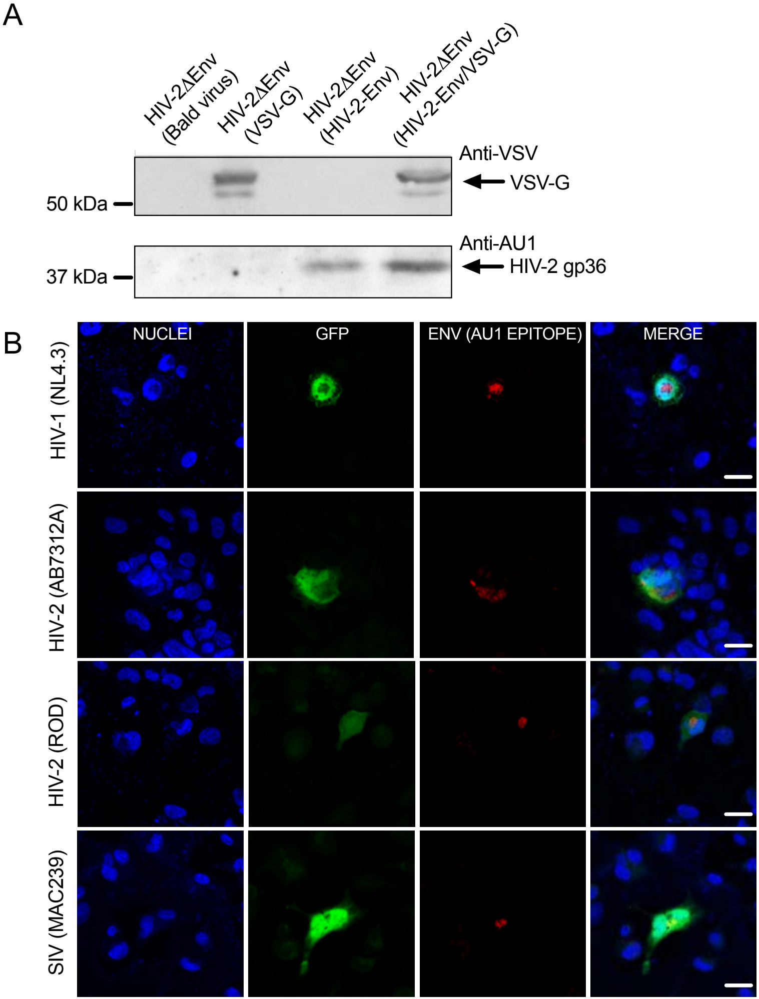
A
HIV-2ΔEnv (Bald virus)
HIV-2ΔEnv (VSV-G)
HIV-2ΔEnv (HIV-2-Env)
HIV-2ΔEnv (HIV-2-Env/VSV-G)
Anti-VSV
VSV-G
50 kDa
Anti-AU1
HIV-2 gp36
37 kDa
B
NUCLEI
GFP
ENV (AU1 EPITOPE)
MERGE
HIV-1 (NL4.3)
HIV-2 (AB7312A)
HIV-2 (ROD)
SIV (MAC239)

Sup Fig 6
